# Regenerative Endodontic Treatment of Maxillary Incisors with a History of Severe Traumatic Injury

**DOI:** 10.1155/2021/4737104

**Published:** 2021-10-08

**Authors:** Nazanin Chitsaz, Mehrfam Khoshkhounejad, Hadi Assadian, Behnam Bolhari, Mohammadreza Sharifian, Zahra Mohammadi

**Affiliations:** Department of Endodontics, School of Dentistry, Tehran University of Medical Sciences (TUMS), Tehran, Iran

## Abstract

The treatment objective for children with avulsed anterior teeth should concentrate on preserving the alveolar bone volume and contour. Posttraumatic external inflammatory root resorption (EIRR) is also a high-risk complication often observed in children. Regenerative endodontic procedure (REP) has been considered a successful treatment to arrest EIRR, especially in posttraumatic cases. This case report presents clinical outcomes of REP in two teeth of an 8-year-old systemically healthy patient with a history of severe dentoalveolar traumatic injury, one with a history of avulsion, and the other with an EIRR. The treatment consisted of REP on both teeth #8 and #9. The results showed some evidence of maturation in the apical third of tooth #9 and resolution of signs and symptoms on tooth #8.

## 1. Introduction

Dental trauma is a significant risk to dental health in young individuals. Dental trauma often occurs in children between 6 to 13 years of age, most commonly involving teeth with incomplete root formation. The most frequent dental injury at this age is crown fracture [1]. Avulsion of permanent teeth is the most severe dentoalveolar injury. It is relatively infrequent, ranging from 0.5% to 16% of traumatic injuries in the permanent dentition [2, 3]. In a literature review, Glendor concluded that one-fourth of all school children and one-third of adults suffered from traumatic dental injuries (TDIs) in permanent dentition [4]. Injuries include fracturing, displacing, or losing teeth, crushing and/or fracturing bone and soft tissue contusions, abrasions, and lacerations [5].

Tooth avulsion, which is also known as exarticulation or total luxation, is defined as complete displacement of the tooth out of its alveolar socket [6]. Loss of permanent teeth in growing children can result in severe atrophy of the residual ridge, which is difficult to restore with either implants or fixed partial dentures [5]. Therefore, preservation of the avulsed teeth is of utmost importance especially in anterior region for future reconstruction. Consequently, replantation is currently considered to be the treatment of choice for avulsed permanent teeth, and immediate replantation of the avulsed tooth is the best treatment at the place of the accident. However, common complications such as pulp necrosis and infection, ankylosis-related (replacement) resorption, and infection-related (inflammatory) resorption are frequently seen following replantation of avulsed permanent teeth [5–7]. The International Association for Dental Traumatology outlined guidelines advocating that replantation should be performed within 60 minutes after injury to have a better prognosis. Delayed replantation (>60 minutes) has been shown to have a poor long-term prognosis with eventual ankylosis and resorption [5, 8, 9]. Delayed replantation is preferred over not replanting in children with avulsed teeth because promotion of alveolar bone growth will be more predictable. In addition, replantation can provide improved esthetics, and function and psychological reasons are other benefits of replantation [10]. Following avulsion, the most desirable outcome can be met after a REP since the damaged tooth can pursue its maturation process [11]. It has been shown that delayed replantation of avulsed immature teeth provides a poor prognosis due to the sustained damages to the pulp and periodontal tissues which can impede further radicular development [6].

External root resorption (ERR) is considered a frequent consequence of severe dentoalveolar traumatic injury. EIRR which is a type of ERR is a pathologic process commonly seen after infection, pressure, trauma, or orthodontic tooth movement. In other words, EIRR occurs as a consequence inflammation and loss of radicular protective barriers following mechanical or chemical insults [12]. Posttraumatic EIRR is a considerable and common complication of dentoalveolar traumatic injuries in children [13]. Several treatment options have been proposed by authors to arrest EIRR with contradictory, uncertain, and unpredictable results [14–16]. Lately, Yoshpe et al. [14] described a case in which a regenerative endodontic procedure (REP) using PRF as a scaffold showed arrested EIRR of tooth #9, which had undergone trauma-induced EIRR.

This study is aimed at describing clinical outcomes of REPs in two teeth of a patient with a history of avulsion of tooth #8 and EIRR in tooth #9.

## 2. Case Report

**Figure 1:**
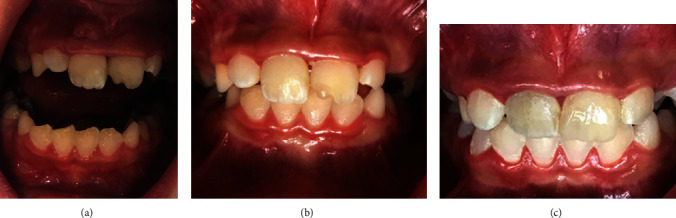
(a) Mucosal sinus tract on the maxillary right central incisor. (b) Four-month follow-up. (c) Fourteen-month follow-up.

An 8-year-old girl was referred to the Department of Endodontics at Tehran University of Medical Sciences (TUMS) School of Dentistry with a chief complaint of a sinus tract in the maxillary right central incisor (Figure 1(a)). She presented normal systemic health and corresponded to class I according to ASA health classification. Patient's dental history revealed a traumatic injury about one month before her referral that had caused avulsion of tooth #8 and complicated crown fracture of tooth #9. On the day of trauma, the patient had been referred to an emergency department of a hospital with a delay of 5 hours, and the tooth had been replanted. Patient's avulsed tooth had been kept in patient's mouth before replantation. Following replantation, a splint was established with sutures. Afterward, the patient was referred to the Department of Pediatric Dentistry the day after the injury for partial pulpotomy of tooth #9. Eventually, the patient was referred to the Department of Endodontics about one month later to evaluate and treat a sinus tract on tooth #8.

**Figure 2:**
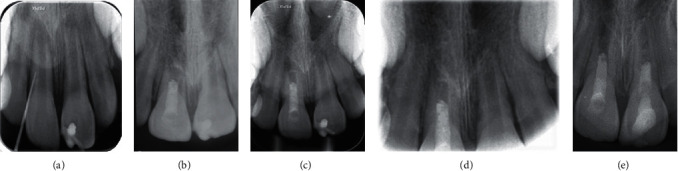
(a) Periapical radiograph of the maxillary right central incisor with traced mucosal sinus tract using a no. 30/0.02 gutta-percha point. (b) Immediate postoperative radiograph showing completion of RET. (c) Two-month follow-up. (d) Four-month follow-up. (e) Fourteen-month follow-up.

Tooth #8 did not respond to pulp sensibility tests. It had grade I mobility and was sensitive to percussion. The mucosal sinus tract was traced with a #30/.02 gutta-percha point, and a periapical radiograph was taken to ensure the origin of infection (Figure 2(a)). Tooth #8 had an open apex, short root, and thin root canal walls. Pulp revascularization treatment was considered regarding root development and apical maturation status. The patient was informed about the treatment process, and informed consent was taken. In the first session, buccal infiltration local anesthesia was administered using 2% lidocaine with 1 : 80,000 epinephrine. The access cavity was prepared under rubber dam isolation. The working length was determined using an electronic apex locator (Root ZX, J. Morita, Tokyo, Japan) and confirmed with a periapical radiograph. Then, chemical disinfection was carried out with use of 1.5% sodium hypochlorite as the irrigating solution with gentle mechanical instrumentation. Double antibiotic paste (DAP) was prepared using equal weight ratios of metronidazole and ciprofloxacin and placed within the prepared root canal after drying. The access cavity was sealed with reinforced zinc oxide eugenol cement (Zoliran, Golchai, Iran). Patient evaluation in the second treatment session, three weeks later, indicated complete resolution of the preexisting symptoms and healing of the sinus tract. In the second treatment session, after administering local anesthesia using infiltration of 3% mepivacaine without a vasoconstrictor, the tooth was isolated with a rubber dam. Intracanal medication was then removed with 20 mL of normal saline solution. Irrigation was carried out using 20 mL of 17% EDTA solution for 5 min. The root canal was then dried with sterile paper points. A blood sample was taken from the patient, and platelet-rich fibrin (PRF) was prepared using a duo centrifuge (Process for PRF, Nice, France). Afterward, bleeding was induced using a sterile #30 hand K-file (Mani, Japan) beyond the apex. The prepared PRF was placed within the coronal part of the root canal 3 mm apical to the cementoenamel junction (CEJ). Then, a light-cured dentin-bonding agent (Solobond Plus, DMG, Hamburg, Germany) was applied to the labial surface of the pulp chamber to seal off the dentinal tubules of the crown. Ortho-MTA (BioMTA, Daejon, Seoul, Korea) was used as the coronal barrier, and the access cavity was temporarily filled with reinforced zinc oxide eugenol cement (Zoliran, Golchai, Iran) after placing a moist cotton pellet on the coronal barrier.

The final setting of the OrthoMTA as the coronal barrier material was confirmed four days later, and the tooth was permanently restored using Filtek Z350 (3M ESPE) nanocomposite material after applying a thin layer of restorative light-cured glass-ionomer cement (GC Fuji II LC, GC, Tokyo, Japan) (Figure 2(b)). At 2-month (Figure 2(c)) and 4-month (Figures 1(b) and 2(d)) recall visits, tooth #8 showed no significant discoloration or mobility. The probing depths were within normal range, with no sensitivity to percussion or palpation. At the 2-month recall, tooth #9 responded positively to pulpal sensibility tests, but it was slightly sensitive to percussion. Probing depth and mobility were within the normal range. At the 4-month recall, it was sensitive to percussion and showed evidence of external root resorption. Therefore, a CBCT examination was ordered for a thorough three-dimensional evaluation of the resorptive defect.

**Figure 3:**
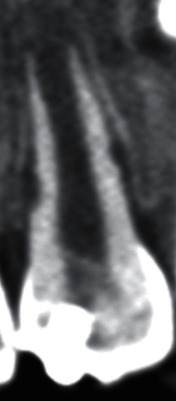
Coronally sectioned CBCT image indicating evidence for external root resorption.

According to CBCT (Figure 3) and evidence of external root resorption, a new treatment plan was scheduled for tooth #9. Since it had an open apex, short roots, and thin walls, pulp revascularization treatment was considered using the same procedure as described above for tooth #8.

At 14-month recall (Figures 1(c) and 2(e)) (10 months after initial regenerative endodontic treatment of tooth #9), teeth #8 and #9 were asymptomatic. They showed no sensitivity to percussion and palpation. Probing depth and mobility were within the normal range. External root resorption at tooth #9 was arrested. Both teeth showed evidence of tooth discoloration. Radiographic examination showed open apex in tooth #8 and little evidence of apical closure in tooth #9.

## 3. Discussion

Traditionally, necrotic traumatized immature permanent teeth have been treated with either long-term calcium hydroxide or MTA apical plug; nevertheless, these methods have a number of disadvantages. One of the disadvantages of calcium hydroxide apexification is the possibility of higher tooth fragility due to long-term use of calcium hydroxide and long period of treatment. Disadvantages of MTA apical plugs include lack of root canal development and the possibility of further tooth fracture due to thin root walls. Another treatment option in these cases is regenerative endodontic procedures (REPs), with benefits such as the possibility of tooth root development and the possibility of increasing the thickness of the root canal walls [17, 18]. For the same benefits, REP was used in this case report of necrotic immature teeth of a patient with history of dental trauma (complicated crown fracture and avulsion).

A recent study revealed that the root development potential of immature necrotic teeth is related to the vitality of Hertwig's epithelial root sheath (HERS) [15]. Lack of continuous root development in the present case could be attributed to the damage to HERS during the traumatic injury. This case had a history of avulsion in tooth #8, considered the most severe dental injury with an unpredictable prognosis [16]. Therefore, we do not expect root development in tooth #8, while there is a higher probability of root development in tooth #9 with a history of complicated crown fracture trauma. In this case in tooth #8, no increase in root length or apex closure was observed in a 14-month follow-up, while tooth #9 with a history of complicated crown fracture showed evidence of apex closure on periapical radiography.

The long-term prognosis of replanted teeth is usually influenced by the extra-alveolar time of storage and the medium used, which are crucial in significantly increasing the viability of periodontal ligament cells and cementum [19, 20]. The International Association for Dental Traumatology (IADT) recommends that avulsed teeth be replanted immediately [5, 19].

There is a possibility of revascularization of permanent immature teeth in dry time less than an hour (IADT) [5]. In one study, it has been shown that replantation within 45 minutes increases the chance of revascularization [18].

Although it has been shown that saliva may not be the most suitable medium for long-time avulsed teeth, it can serve as a short-term storage medium for more than an hour for PDL cells. However, storage for 2 to 3 hours causes swelling and damage to the membrane of PDL cells and is also not desirable due to the presence of microorganisms in the saliva of the storage medium [21]. In this case, tooth #8 had been kept in patient's mouth for 5 hours before replantation. Therefore, prolonged extraoral time and inappropriate storage medium will be responsible for its compromised prognosis.

External root resorption (ERR) is often a consequence of a severe traumatic injury to dental structures. The presence of pulp necrosis combined with trauma to the tooth may have led to external inflammatory resorption of the root [22]. Long-term calcium hydroxide has been used for treatment of external root resorption. Calcium hydroxide can change the acidic environment of the resorbed root surface and prevent osteoclasts and odontoclasts from functioning. Calcium hydroxide may also be able to induce hard tissue. Recently, regenerative therapy has been used to manage the external root resorption of traumatized teeth. The resorption includes dentin and cementum, which is repaired by new cementum due to the presence of periodontal ligament stem cells, which, if properly signaled, can differentiate into cementoblast, not odontoblast, and repair the area with cementum [17].

In this case, tooth #9 showed external root resorption in the coronal third of the root, which was arrested after RET, and a normal periodontal ligament was seen around the area of arrested resorption at 10-month follow-up. However, longer follow-up is recommended. Likewise, Yoshpe et al. reported that REP is a promising treatment modality for arresting ERR [14]. Lu et al. showed that REP might be used to manage traumatized immature permanent teeth with necrotic pulp and apical periodontitis associated with severe external root resorption and root perforation. It was also shown that the resorptive area could be arrested and subsequently be repaired with hard tissue [17]. Dastpak et al. performed regenerative endodontic procedures for necrotic immature upper central incisor with external root resorption and chronic apical periodontitis in a patient with history of trauma. In a 12-month follow-up, the tooth was completely asymptomatic, and crown discoloration was observed. This case showed that REP can be effective in arresting ERR [23]. Giorgio et al. reported management of a case with external root resorption in an immature tooth #8 with a history of avulsion and presence of complicated crown fracture in a necrotic tooth #9. Apexification treatment by MTA apical plug was performed for both teeth. A 24-month follow-up revealed arresting root resorption in tooth #8, as well as ankylosis [24].

One serious complication after tooth avulsion and reimplantation is replacement root resorption (RRR) (ankylosis). The risk of ankylosis increases with prolonged period of dry time [24, 25, 5]. However, there is no definitive treatment for arresting RRR. According to Yoshpe et al., RET using PRF has the potential to arrest RRR [14]. Anyway, if ankylosis occurred, and there was evidence of intolerable infraocclusion from esthetic point of view that cannot be managed by restorative material, decoronation is needed [24]. Preservation of bone height and width following decoronation was observed [26].

Tooth discoloration is a common complication of RET [27]. This discoloration can be due to three reasons: intracanal drug, intracanal bleeding, and biomaterials. The intracanal medicament minocycline in triple antibiotics paste is responsible for discoloration [28], which, of course, was not used in this case, and DAP was used. Tripathi et al. reported that coronal tooth discoloration occurred after using white MTA as a coronal barrier in RET [29]. Additionally, in this case, teeth #8 and #9 showed minor discolorations at the 14-month follow-up, although the resolution of symptoms and restoration of dental functions happened following RET. Discoloration in the present case can be due to both bleeding and biomaterial. To prevent this, the use of bonding is recommended [30, 31].

American Association of Endodontics (AAE) [30] categorized success rates of RETs as primary, secondary, and tertiary goals, with elimination of symptoms and bone healing as primary, root maturation as secondary, and positive response to vitality testing as the tertiary goals. European Society of Endodontology (ESE) [31] describes success of regenerative endodontic procedure as lack of signs and symptoms, discoloration, and patient's acceptance and radiographic detection of new PDL along the inner wall of root canal.

From this point of view, tooth #8 belongs to “primary success” and tooth #9 belongs to “secondary success” according to AAE [30]. However, taking the ESE guidelines into consideration, the treatment was considered as “failed” due to the lack of root length increase and discoloration [31]. Therefore, AAE criteria for success in RETs seems to be a more comprehensive, more specifically in border line cases.

The current case report indicated that continued root development and increase in root canal wall thickness are not mere success criteria for RET. However, tooth functionality and esthetics, especially for young patients and supporting bone growth through the skeletal maturation period, should also be considered as further criteria for success. If the involved tooth becomes symptomatic in the future, other treatment options such as using dental implants in the defective site will be considered to restore patient's esthetics and function.

## Data Availability

Almost all data is given and reviewed in the article, and if more data is needed, it will be sent to you from the corresponding author upon request.
